# Effect of Nitrogen, Air, and Oxygen on the Kinetic Stability of NAD(P)H Oxidase Exposed to a Gas–Liquid Interface

**DOI:** 10.1021/acs.oprd.3c00095

**Published:** 2023-05-19

**Authors:** Jingyu Wang, Elif Erdem, John M. Woodley

**Affiliations:** Department of Chemical and Biochemical Engineering, Technical University of Denmark, 2800 Kgs. Lyngby, Denmark

**Keywords:** enzyme stability, NAD(P)H oxidase, gas−liquid interface, nitrogen, air, oxygen

## Abstract

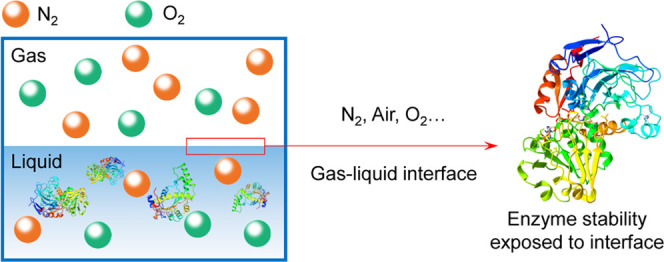

Biocatalytic oxidation is an interesting prospect for the selective synthesis of active pharmaceutical intermediates. Bubbling air or oxygen is considered as an efficient method to increase the gas–liquid interface and thereby enhance oxygen transfer. However, the enzyme is deactivated in this process and needs to be further studied and understood to accelerate the implementation of oxidative biocatalysis in larger production processes. This paper reports data on the stability of NAD(P)H oxidase (NOX) when exposed to different gas–liquid interfaces introduced by N_2_ (0% oxygen), air (21% oxygen), and O_2_ (100% oxygen) in a bubble column. A pH increase was observed during gas bubbling, with the highest increase occurring under air bubbling from 6.28 to 7.40 after 60 h at a gas flow rate of 0.15 L min^–1^. The kinetic stability of NOX was studied under N_2_, air, and O_2_ bubbling by measuring the residual activity, the deactivation constants (*k*_d1_) were 0.2972, 0.0244, and 0.0346 with the corresponding half-lives of 2.2, 28.6, and 20.2 h, respectively. A decrease in protein concentration of the NOX solution was also observed and was attributed to likely enzyme aggregation at the gas–liquid interface. Most aggregation occurred at the air–water interface and decreased greatly from 100 to 14.16% after 60 h of bubbling air. Furthermore, the effect of the gas–liquid interface and the dissolved gas on the NOX deactivation process was also studied by bubbling N_2_ and O_2_ alternately. It was found that the N_2_–water interface and O_2_–water interface both had minor effects on the protein concentration decrease compared with the air–water interface, whilst the dissolved N_2_ in water caused serious deactivation of NOX. This was attributed not only to the NOX unfolding and aggregation at the interface but also to the N_2_ occupying the oxygen channel of the enzyme and the resultant inaccessibility of dissolved O_2_ to the active site of NOX. These results shed light on the enzyme deactivation process and might further inspire bioreactor operation and enzyme engineering to improve biocatalyst performance.

## Introduction

**Scheme 1:**
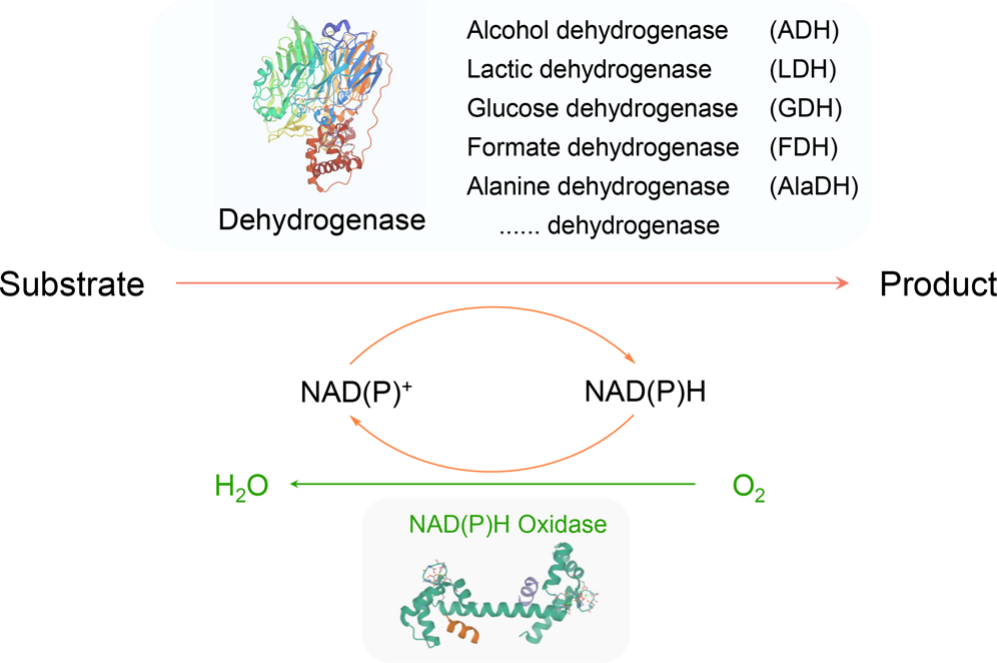
Biocatalytic Oxidation by a Dehydrogenase Coupled with In Situ Cofactor Regeneration Using Water-Forming NOX

Biocatalytic oxidation is currently receiving great interest as a tool in synthetic and industrial chemistry with high selectivity compared with traditional catalysis.^1−3^ For example, alcohol dehydrogenase (ADH) has been used to oxidize lactols to chiral intermediate lactones together with NAD(P)H oxidase (NOX) for cofactor regeneration on a 100 L scale.^4^ Likewise, a renewable and sustainable route for polymer production based on 2,5-furandicarboxylic acid (FDCA) can be realized via the oxidation of biobased 5-hydroxymethylfurfural (HMF) using galactose oxidase (GOase),^5,6^ which would be an ideal way to utilize renewable biomass for sustainability.^7^ Generally, monooxygenases and oxidases have been developed for biocatalytic oxidation processes, and oxygen has been supplied as an electron acceptor (oxidant) with the ensuing H_2_O or H_2_O_2_ -associated byproducts.^8^ Additionally, peroxygenases and dioxygenases have also been developed (although to a lesser extent), which makes the toolbox of the biocatalytic oxidation process even more diverse, but enzyme engineering is still required for enzyme activity and stability improvements and developing a broad substrate scope.^9,10^ However, dehydrogenases are still attractive and relatively well developed with enhanced activity and thermostability.^11^ They are routinely used for reduction but can also be applied in biocatalytic oxidation processes together with the nicotinamide cofactor NAD(P)^+^ as an electron acceptor. Likewise, (*R*)-undecavertol (>98%, *ee*) was produced by (*S*)-selective ADH alcohol dehydrogenase with a 400 g L^–1^ substrate concentration on a pilot scale.^12^ Meanwhile, the cofactor NAD(P)^+^ can be regenerated *in situ* by NOX for economic sustainability.^13^ During this reaction, oxygen is generally considered as the most suitable oxidant since it is economical and readily available in air, with water as the only byproduct (Scheme 1).

Such a biocatalytic oxidation route would seem to be an ideal process for industry, the oxygen from air is the ideal oxidant for the biocatalytic oxidation process, while pure oxygen can be supplied to promote the oxygen mass transfer in industries where this is allowed.^4,13^ However, the effect of pure oxygen in the gas phase on enzyme stability is not known and requires further study. In previous work, it was found that the ADH/NOX was more stable with a low oxygen gas flow rate and resulted in a high product conversion to produce (*R*)-undecavertol with a pure oxygen supply, achieving a space–time yield of 14 g L^–1^ h^–1^.^12^ In another report, it was found that the stability of l-amino acid oxidase was affected by the dissolved oxygen concentration and that the reaction rate and productivity were increased by fast and continuous bubbling with air.^14^ In addition, it turns out that the affinity constant of an oxidase toward oxygen (*K*_MO_) is also an important factor that determines the way the oxygen should be supplied.^5,15,16^ If the *K*_MO_ of an enzyme is low, air supply might be adequate to reach the maximum activity, and the dissolved oxygen is not limiting.^17^ For example, the initial reaction rate of a lipoxygenase to convert linoleic acid into linoleic acid hydroperoxide has no significant decrease when the initial dissolved oxygen concentration was adjusted from 10 to 80% of saturation, which indicates a low *K*_MO_ for lipoxygenase.^18^ Therefore, the increasing oxygen concentration may not result in high enzyme activity if the *K*_MO_ of an oxidase is low, and the high activity and stability can be achieved through protein engineering alone.^19,20^ However, a gas phase with high oxygen concentration may decrease the reaction rate because of fast enzyme deactivation.^21^ It was also reported that hydrogenases are sensitive to oxygen concentration since their activity decreases as the oxygen partial pressure increases.^22,23^ Therefore, an oxidant with air or pure oxygen is a critical issue that may have different effects on enzyme activity and stability. Further study is required to understand and guide the application of biocatalytic oxidation in industry.

In one study, in a controlled bubble column, we previously reported that NOX deactivation was related to the residence time of the gas–liquid interface, the long half-life of NOX under fast gas bubbling was attributed to the low residence time of bubbles in the bubble column.^13^ On this basis, we herein further investigate the effect of gas on the kinetic stability of NOX when bubbling different gases. The effect of N_2_, air, and O_2_ on NOX stability was studied by measuring the residual activity of NOX after different gas bubbling times. The operation conditions of a running bubble column (pH, temperature, and dissolved O_2_ concentration) were also measured online by relevant sensors. The protein concentration of NOX solution was also evaluated during the experiments, and the corresponding specific activity of NOX was calculated to further understand and distinguish the effect of the dissolved gas and gas–liquid interface on the kinetic deactivation. Structural analysis of NOX was also carried out to help explain the NOX deactivation process at gas–liquid interface. This study was intended to help understand the enzyme deactivation process at a gas–liquid interface and provide inspiration for protein engineering to further improve the NOX performance under process conditions.

## Experimental Section

### Materials

NAD(P)H oxidase (PRO-NOX 001) was supplied by Prozomix Limited (Haltwhistle, Northumberland, UK). NADPH was purchased from Bontac Bio-Engineering (Shenzhen, Guangdong, China). Bovine serum albumin (BSA, pH 7, ≥98%), KH_2_PO_4_, and K_2_HPO_4_ were purchased from Sigma-Aldrich (St. Louis, MO). The Coomassie Plus protein assay reagent was purchased from Thermo Fisher Scientific (Waltham, MA). All chemical reagents used in experiments were of analytical grade and used directly without any further purification.

### Experimental Procedure

#### Enzyme Activity Assay

The activity assay and kinetic stability experiments for NOX have been described in our previous study.^13^ In detail, a NOX solution (500 μL, 0.05 g L^–1^) was mixed together with NADPH (1 g L^–1^, 100 μL) and phosphate buffer (50 mM, pH 7.0, 400 μL) in a semi-micro cuvette, which was shaken using a mixer (ZX3 Advanced Vortex Mixer, Usmate Velate, Italy) at 1600 rpm at 25 °C. Then, the sample was measured by a UV–vis spectrophotometer (Shimadzu UV-1800, Kyoto, Japan) for 2 min, and the decreasing rate of NADPH adsorption at 340 nm was correlated with the activity of NOX. The corresponding extinction coefficient was ε_340_ = 6.22 mM^–1^ cm^–1^. One unit (U) of NOX activity was defined as 1 mM NADP^+^ produced per minute at 25 °C, pH 7.0. The liquid level of NOX solution in bubble column was marked and replenished with deionized water in time due to the water evaporation caused by the continuous gas bubbling.

#### Kinetic Stability of NOX

**Figure 1:**
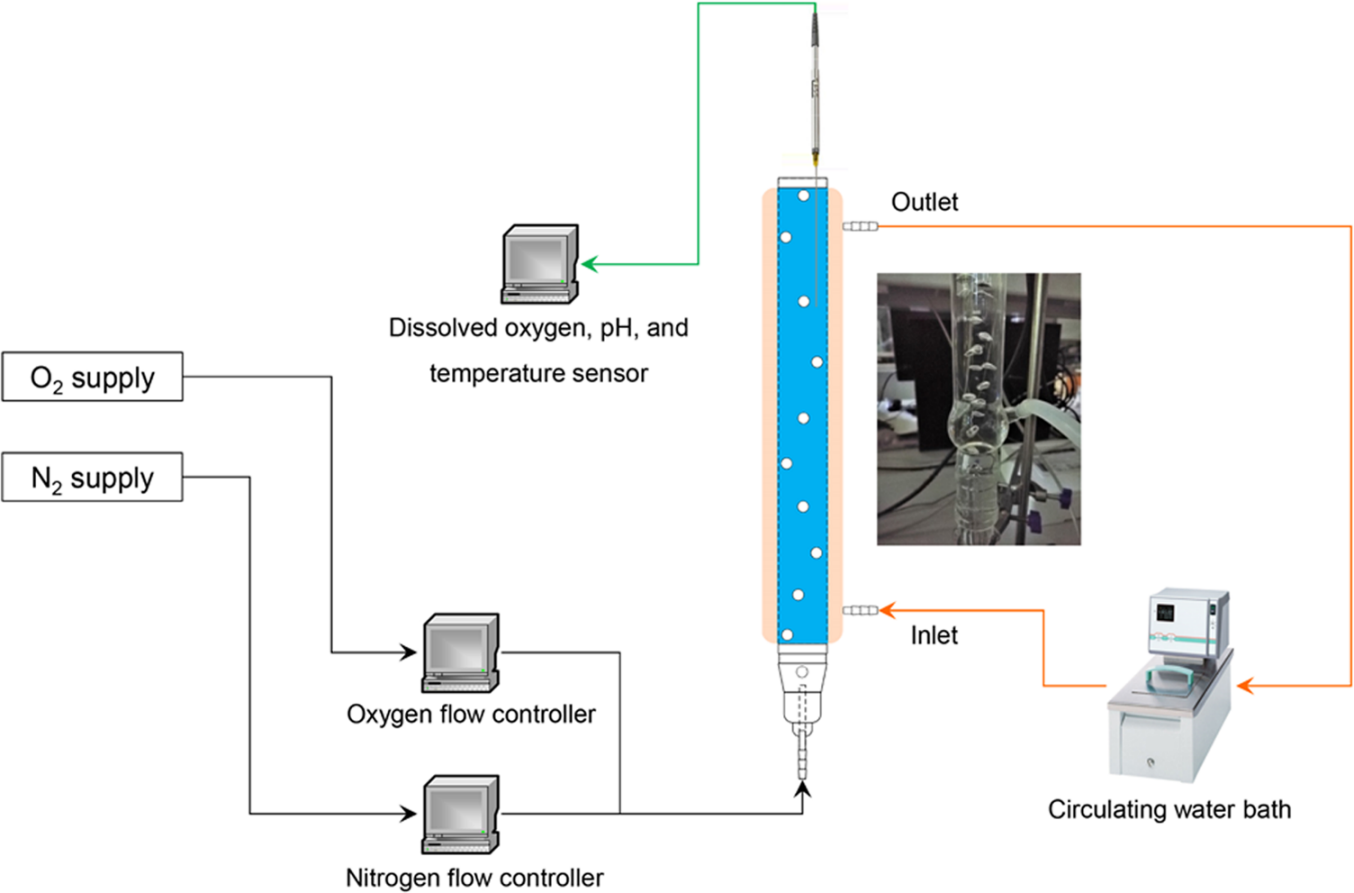
Bubble column set-up for controlled exposure of NOX.

A NOX solution (200 mL, 0.05 g L^–1^) was incubated in a bubble column to study the kinetic stability under various gases and rates of bubbling (Figure 1). The gas flow rate was controlled by an oxygen flow meter and a nitrogen flow meter (Sierra SmartTrak 50, Monterey, CA) together with SmartTrak 50 product software (S50 firmware, *version* 1.05). Samples were taken during gas bubbling to measure the residual activity of NOX (Figure S1). The protein concentration was measured by the Coomassie Plus protein assay reagent at a ratio of 1:1 with NOX solution and measured at 595 nm using a UV–vis spectrophotometer (Figure S2).

#### Bubble Column Data Collection

In our previous work, the gas bubble shape and the area of the gas–liquid interface have been described.^13^ Here, the pH, temperature, and dissolved O_2_ concentration in the enzyme solution of the bubble column were monitored online by the Pyro Workbench connected to sensors FireSting-PRO (4 Channels, PyroScience GmbH, Aachen, Germany). The pH sensor was calibrated with the standard solution at pH 2 and 11 before use. The pH values of deionized water and the NOX solution before and after gas bubbling were measured by a PHM220 MeterLab pH meter (Radiometer Analytical, Lyon, France). The pH meter was calibrated with a standard solution at pH 7 and 10 before use.

#### Calculation Methods

The residual activity of NOX was fitted with a first-order deactivation kinetic experiment to calculate the deactivation kinetics
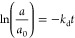
1where *a* and *a*_0_ are the enzyme activity at *t =* 0 h and *t* h, and *k*_d_ is the enzyme deactivation kinetic constant. *k*_d1_ and *k*_d2_ are also introduced to represent the non-first-order degradation over the entire degradation. The two constants represent the relevant deactivation constants for the first and second stages.

The dissolved gas concentration of N_2_, air, and O_2_ are calculated by Henry’s law.
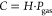
2where *C* is the solubility of a gas in a solvent, *H* is Henry’s law constant, and *P*_gas_ is the partial pressure of the gas.

## Results and Discussion

### Online Monitoring of NOX Solution during Gas Bubbling

**Figure 2:**
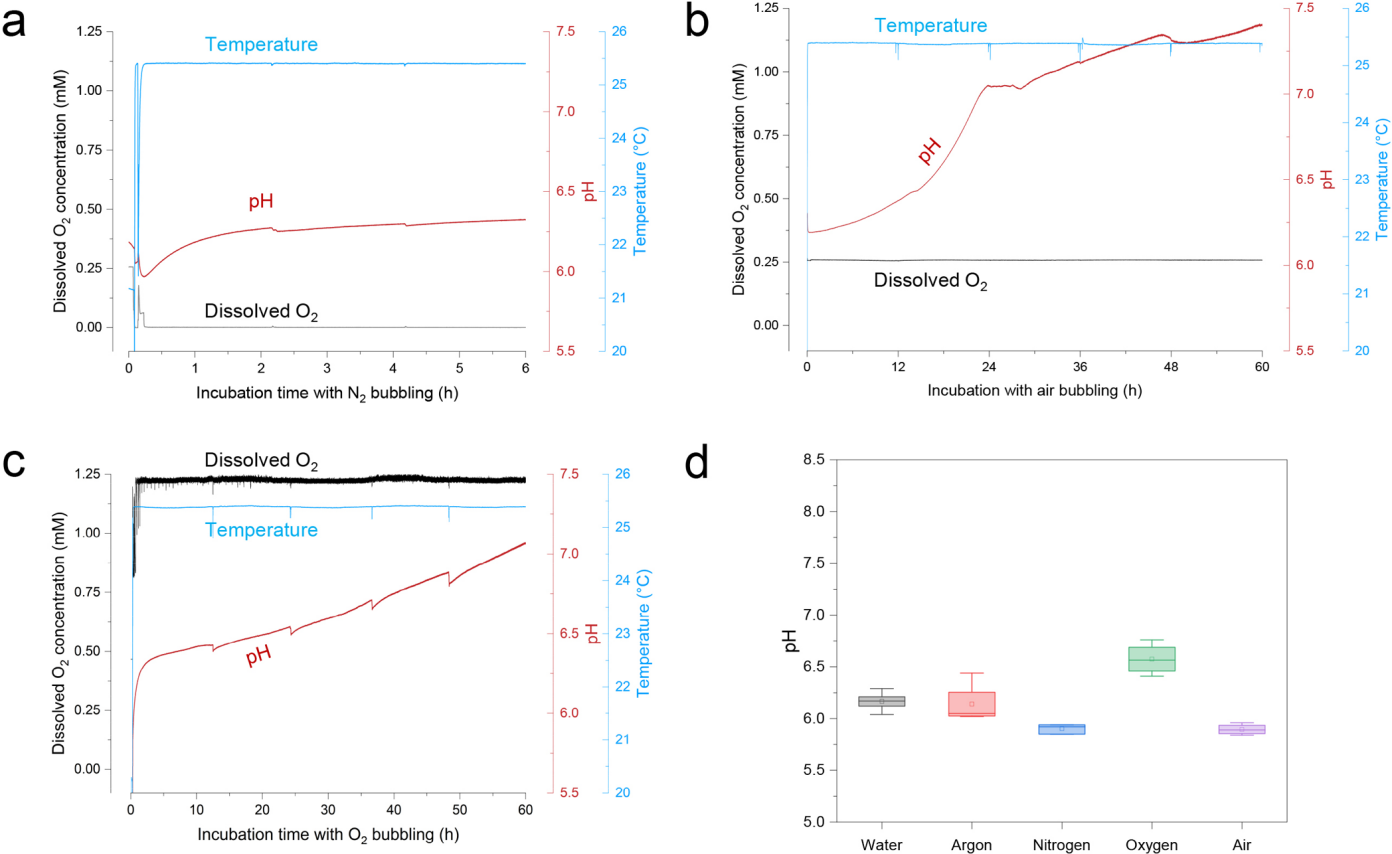
Change of temperature, pH, and the dissolved oxygen concentration of NOX solution in the bubble column as N_2_ (a), air (b), and O_2_ (c) were supplied for gas bubbling. (d) pH change of deionized water after gas bubbling (control experiment); 25 °C, 200 mL of NOX solution, and 0.15 L min^–1^ gas flow rate.

The concentration of dissolved O_2_ is critical for most biocatalytic oxidation processes, as also the pH and temperature of incubation. Therefore, the online conditions such as temperature, pH, and dissolved O_2_ sensors of NOX solution incubated in the bubble column were monitored during gas bubbling, and the results are shown in Figure 2. The temperature of the bubble column was controlled by a circular water bath for 25 °C during all experiments. The equilibrium between dissolved O_2_ and the O_2_ in the gas phase increased quickly according to the results in Figure 2, and likewise, the dissolved O_2_ could be removed very quickly and finally reached zero with N_2_ bubbling (Figure 2a). With gas bubbling, the dissolved O_2_ concentration was maintained constant at 0, 21, and 100% with N_2_, air, and O_2_ bubbling, respectively (Figure 2a–c). The air-saturated water was a 21% O_2_ concentration in the gas phase that coincided with the dissolved O_2_ concentration of 0.26 mM. The pH increase of NOX solution was also found during gas bubbling. In Figure 2a, the pH increased from 6.04 to 6.38 after 6 h of N_2_ bubbling. With O_2_ bubbling, it increased from 6.12 to 7.02 after 60 h of O_2_ bubbling. The dissolved O_2_ concentration was 1.24 mM, where the dissolved O_2_ concentration was 5-fold higher than that in air-saturated water (Figure 2c). It is worth mentioning that the highest pH increase was observed with air bubbling, and it increased from 6.28 to 7.40 after 60 h of bubbling with 0.15 L min^–1^ gas flow rate (Figure 2b). As a control experiment (Figure 2d), the pH of deionized water was little changed after 60 h of gas bubbling with the same gas flow rate of 0.15 L min^–1^. Therefore, the pH increase of NOX solution during gas bubbling might only be attributed to the deactivation of NOX. It has been reported that the bubbles in deionized water were negatively charged, and the gas–liquid interface charge was independent of the bubbling gas.^24^ Therefore, the pH change was only caused by the NOX deactivation process, and the difference of pH change in Figure 2a–c was attributed to the effect of different gas–liquid interfaces on the enzyme deactivation process. Meanwhile, the enzyme might be regarded as a surfactant and contained both hydrophilic and hydrophobic residues, and therefore was adsorbed at the gas–liquid interface. The increasing pH was also related to a decreasing ζ potential of the gas–liquid interface, which might also relate to enzyme unfolding and then its adsorbing at the interface.

### NOX Kinetic Stability under N_2_, Air, and O_2_ Bubbling

#### Effect of Different Gases

**Figure 3:**
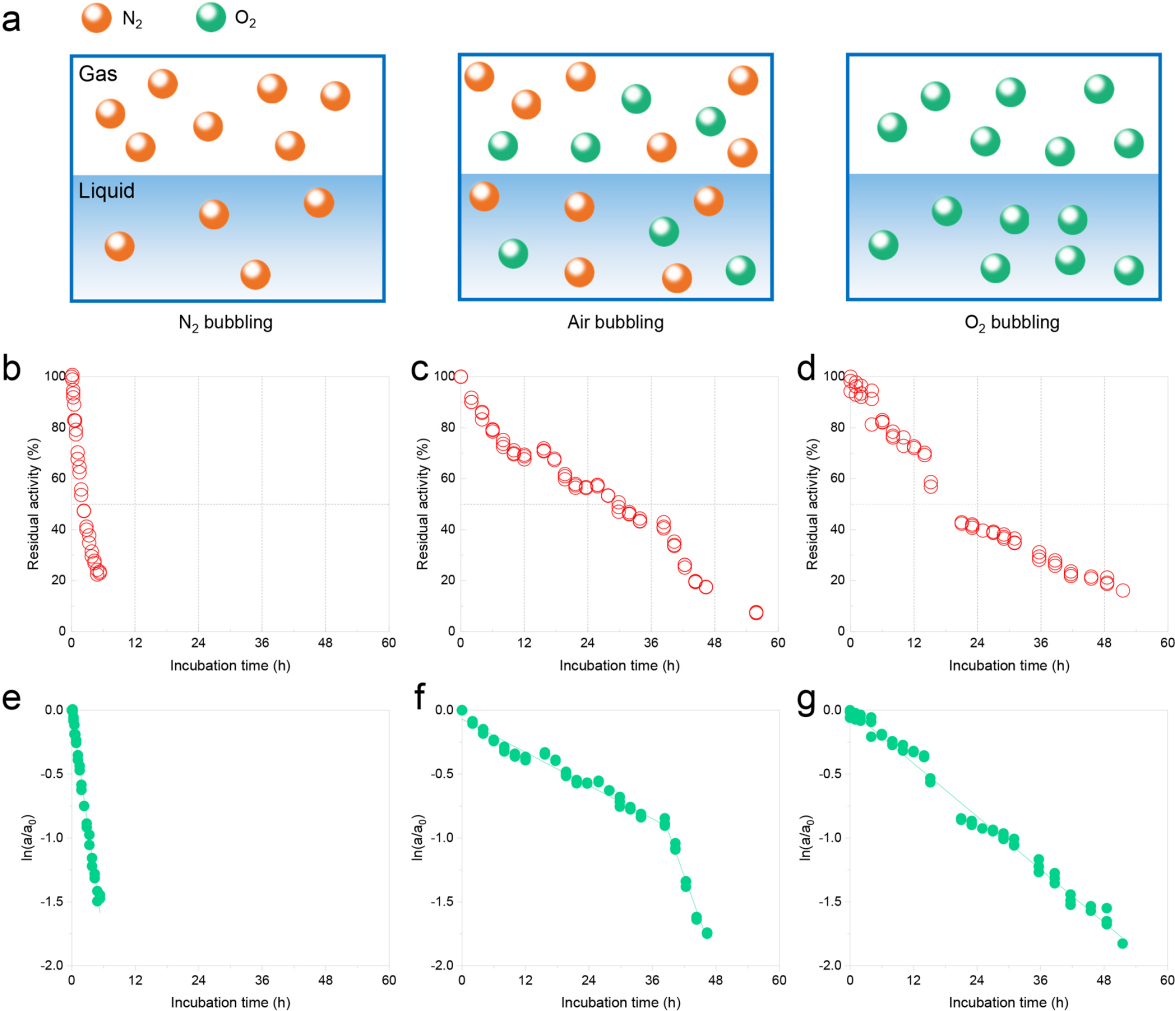
Diagrams of the gas–liquid interface under N_2_, air, and O_2_ bubbling (a) and the corresponding deactivation kinetics of NOX with 0.05 L min^–1^ (b) N_2_, (c) air, and (d) O_2_ gas flow rate of 0.05 g L^–1^ and 200 mL of NOX solution. Deactivation kinetics of NOX with the logarithm fitted equation (e) N_2_ bubbling, *y* = −0.2972*x*, *R*^2^ = 0.9965, single-stage deactivation; (f) air bubbling, first-stage: *y* = −0.0244*x*, *R*^2^ = 0.9874; second-stage: *y* = −0.1150*x* + 3.5337, *R*^2^ = 0.9720; and two-stage deactivation; and (g) O_2_ bubbling, *y* = −0.0346*x*, *R*^2^ = 0.9965, single-stage deactivation.

In another experiment, the NOX kinetic stability was investigated in a bubble column under 0.05 L min^–1^ N_2_, air, or O_2_ gas bubbling with 200 mL of 0.05 g L^–1^ NOX solution at 25 °C (Figure 3). A schematic diagram of the gas–liquid interface (Figure 3a) illustrates the continuously and constantly generated gas–liquid interface by gas bubbling, which promotes the mass transfer of oxygen from the gas to the liquid phase effectively. With a controlled gas bubbling rate, the gas–liquid interfacial area was easy to calculate compared with a stirred tank reactor and thereby investigate the effect of the gas–liquid interface on the enzyme stability. The ratio between the NOX concentration and the gas–liquid interface area (*E*_ratio_) was used to describe the operating conditions in a bubble column reactor.^13^ The kinetic stability of NOX was studied under N_2_, air, and O_2_ bubbling by measuring the residual activity; the deactivation constants were 0.2972, 0.0244, and 0.0346 (*k*_d1_) with the corresponding half-lives of 2.2, 28.6, and 20.2 h (Table 1). Two-stage deactivation kinetics was found when NOX was exposed to the air–water interface with 0.05 L min^–1^ gas flow rate, and the *k*_d1_ and *k*_d2_ were 0.0244 and 0.1150, respectively. It was obvious that the deactivation constant in the second stage (*k*_d2_) was much higher than in the first stage (*k*_d1_). However, single-stage deactivation kinetics was both found when NOX was exposed to N_2_ and O_2_ bubbling with the same gas flow rate. The *k*_d_ of N_2_ bubbling was 0.2972, which was 12-fold and 9-fold higher than those with air and O_2_ bubbling, respectively. It has been reported that the deactivation of NOX at the air–water interface is a time-dependent process, and a long residence time of the interface results in a more serious deactivation of NOX.^13^ This implies that N_2_ and O_2_ themselves (dissolved N_2_ and O_2_) have effects on NOX stability other than the interfaces. The residual activity of NOX under N_2_ bubbling dropped sharply from 100 to 23.6% in the first 6 h with a deactivation constant of 0.2972, which was 12-fold higher than under air bubbling (Figure 3b,c), which indicated that NOX was less stable with N_2_ bubbling and N_2_ was harmful to NOX stability. The NOX has the smallest deactivation constant of 0.0244 under air bubbling compared with N_2_ and O_2_ bubbling, and the residual activity decreased at an almost nearly constant rate during the experiment (Table 1 and Figure 3c). The NOX deactivation constant under O_2_ bubbling was 0.0346, which was bigger than under air bubbling (Figure 3d). This result might be caused by the high O_2_ concentration of dissolved O_2_ and also the O_2_–water interface where the amino acid residues of NOX were oxidized, resulting in faster deactivation than under air bubbling.^25^ However, the deactivation of NOX under N_2_ bubbling needed further study because N_2_ is generally considered a nonreactive gas. Two possible factors (the N_2_–water interface and the dissolved N_2_ in water) might be responsible for this result. In the following experiments, the different effects of dissolved gas and the gas–liquid interface on NOX stability were further verified by bubbling N_2_ and O_2_ alternately.

**Table 1:** Solubility of N_2_ and O_2_ in Water and the Deactivation Kinetics of NOX under Different Gases Bubbling, 0.05 L min^–1^ Gas Flow Rate, 200 mL of NOX Solution, and 25 °C

gas	argon	N_2_	air	O_2_
dissolved N_2_ in water (mM)^a^		0.65	0.51	0
dissolved O_2_ in water (mM)^a^		0	0.26	1.24
deactivation process	single-stage	single-stage	two-stage	single-stage
kinetic equation of deactivation	*y = −*0.5715*x*, *R*^2^ = 0.9913	*y = −*0.2817*x*, *R*^2^ = 0.9976	first-stage *y = −*0.0244*x*, *R*^2^ = 0.9874	*y = −*0.0346*x*, *R*^2^ = 0.9965
			second-stage *y = −*0.1150*x* + 3.5337, *R*^2^ = 0.9720	
*k*_d1_ (h^–1^)^b^	0.5715	0.2972	0.0244	0.0346
*k*_d2_ (h^–1^)^b^			0.1150	
half-life (h)^b^	1.14	2.2	28.6	20.2

a Calculated by Henry’s law. The Henry’s law constant of N_2_ and O_2_ at 25 °C were 8.68 × 10^6^ kPa and 4.44 × 10^6^ kPa, respectively; ρ_H_2_O_ = 997.044 g L^–1^ at 25 °C.

b Measured with 0.05 L min^–1^ gas flow rate, 200 mL of NOX solution, and 25 mg L^–1^, 25 °C.

**Figure 4:**
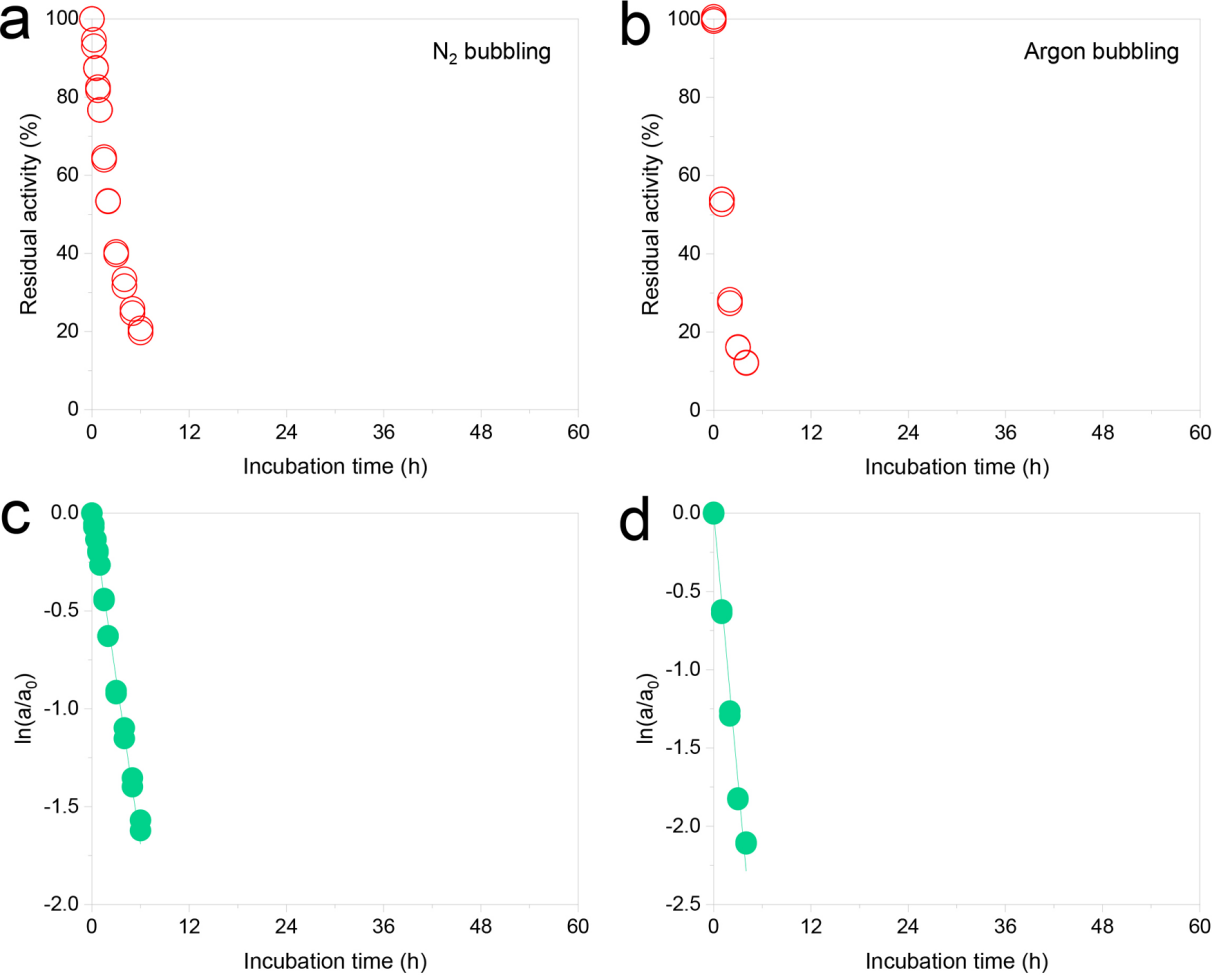
Residual activity of NOX with (a) nitrogen, (b) argon, and the corresponding kinetic stability with the logarithm fitted equation (c) nitrogen, *y* = −0.2817*x*, *R*^2^ = 0.9976; (d) argon, *y* = −0.5715*x*, *R*^2^ = 0.9913 bubbling at 0.15 L min^–1^ gas flow rate, 200 mL, 0.05 g L^–1^ NOX solution, and 25 °C.

Additionally, the effect of argon on NOX stability was investigated and compared with N_2_ bubbling. The results are shown in Figure 4. The deactivation of NOX under argon bubbling was also fast and had a similar decreasing trend to N_2_ bubbling, and the half-life under argon and N_2_ bubbling were 1.14 and 2.2 h, respectively (Figure 4a,b). The deactivation constant *k*_d_ of argon bubbling was 2-fold higher than that for N_2_ bubbling (0.5715 vs 0.2817), suggesting that NOX deactivation occurred more rapidly under argon bubbling (Figure 4c,d). Notably, NOX was much more stable under air bubbling (half-life of 28.6 h) compared with argon and N_2_ bubbling (Figure 3c).

#### Effect of Dissolved Gas and the Gas–Liquid Interface

**Figure 5:**
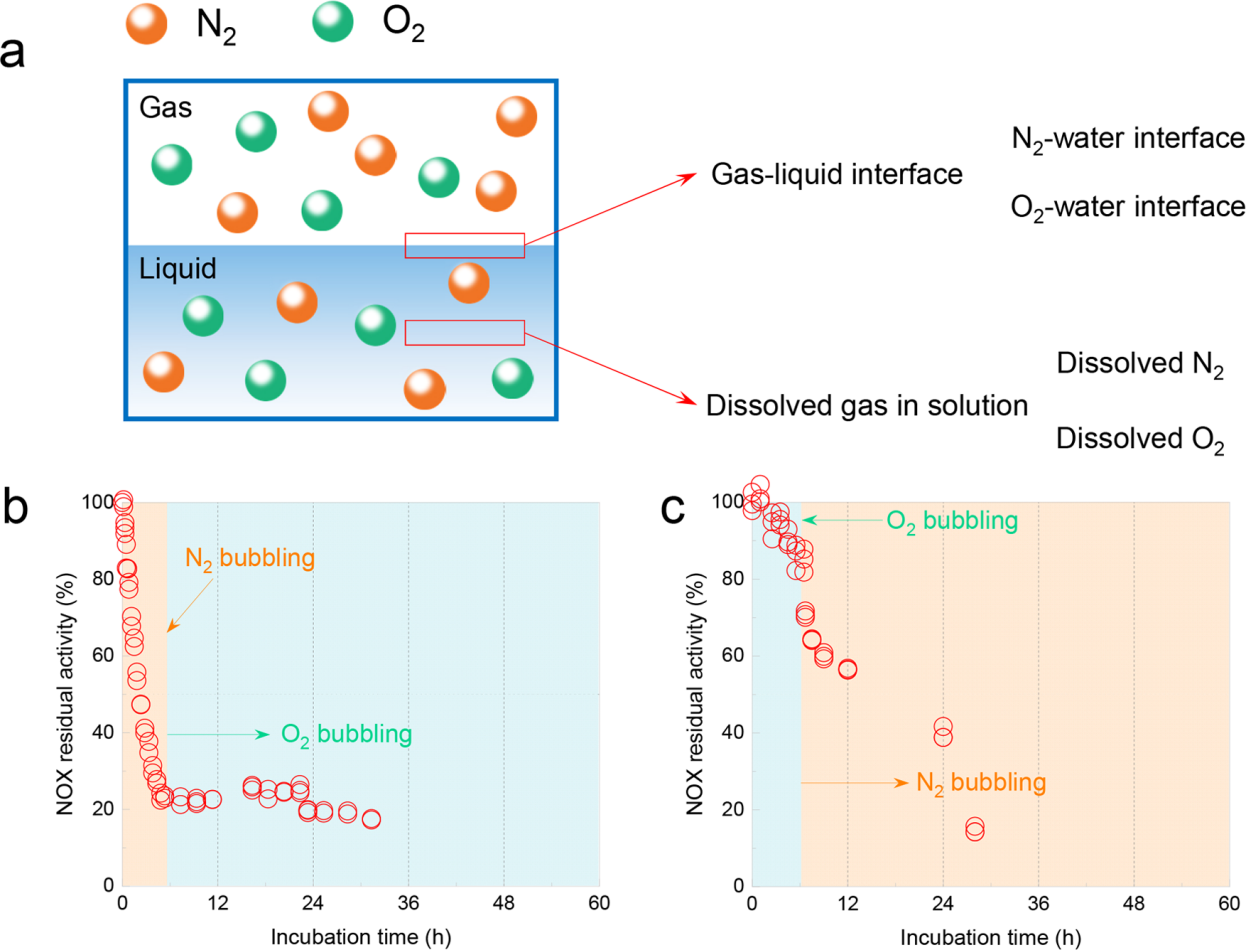
Diagram and description of different gas–liquid interfaces and the dissolved gases’ concentration (a). The residual activity of NOX under N_2_ bubbling 6.5 h and the following O_2_ bubbling (b) and the first O_2_ bubbling 6.5 h and following N_2_ bubbling (c). 0.05 L min^–1^ gas flow rate, 200 mL NOX solution, and 25 °C.

When the enzyme solution was bubbled with gas, the enzyme might be affected by two factors; first, the gas–liquid interface introduced by gas bubbling, and second, the dissolved gas in the aqueous solution (as a result of the mass transfer and phase equilibrium between gas and liquid phases) (Figure 5a). The change of gas phase composition affected the enzyme activity and stability through these two factors. These two effects were further studied in these experiments by first N_2_ bubbling for 6.5 h and then O_2_ bubbling (Figure 5b), and also the inverse way by first O_2_ bubbling for 6.5 h and then N_2_ bubbling (Figure 5c). As shown in Figure 5b, the residual activity of NOX decreased sharply from 100 to 23.3%, with N_2_ bubbling for the first 6.5 h. Subsequently, the NOX activity was not recovered with the following O_2_ bubbling, which indicated that this deactivation of NOX caused by N_2_ bubbling was irreversible (probably because the gas channel of NOX was fully occupied by the dissolved N_2_ first and the following dissolved O_2_ could not enter the active site of NOX anymore). Furthermore, this phenomenon might also be observed in other enzymes containing a gas channel and needing gas as a substrate (e.g., glucose oxidase, galactose oxidase, lipoxygenase). Potentially, if the gas channel was occupied by another nonsubstrate gas, the enzyme activity would lose rapidly and irreversibly. It has been reported that the oxidation performance of oxygenase could be improved by oxygen channel engineering.^20^ In Figure 5c, NOX was first bubbled with O_2_ for 6.5 h, and the NOX activity decreased from 100 to 85%. Following N_2_ bubbling, the NOX activity decreased slowly from 85 to 14% after 28 h, and the activity loss was notably slower compared with Figure 5b, which might indicate that the oxygen channel of NOX was first occupied and saturated by O_2_; therefore, the dissolved N_2_ would not take over the active site of NOX, and only the N_2_–water interface affected the deactivation of NOX.

In Figure 5b,c, it can be concluded that the dissolved N_2_ had a significant effect on NOX activity compared with the N_2_–water interface. The deactivation rate of NOX residual activity in Figure 5c when N_2_ was bubbled (after 6.5 h) was lower than that of the first 6.5 h of N_2_ bubbling in Figure 5b. In addition, both dissolved O_2_ and the O_2_–water interface had a small effect on NOX activity from Figures 3b and 5c. Therefore, the gas channel should be carefully considered since it may be very sensitive and might lose activity when nonsubstrate gases are present.

Furthermore, the dissolved N_2_ and O_2_ concentrations were calculated by Henry’s law, and the results are listed in Table 1. With N_2_ bubbling, NOX was affected by the N_2_–water interface and the dissolved N_2_ (0.65 mM, 25 °C, 1 atm) at the same time. In contrast, NOX was affected by the O_2_–water interface and dissolved O_2_ (1.24 mM, 25 °C, 1 atm). With air or N_2_ bubbling, the dissolved N_2_ concentration was always higher than the dissolved O_2_ concentration. Oxygen might have the advantage of preferentially diffusing into the oxygen channels.^26,27^ According to these results, Figure 5b,c, the dissolved N_2_ was more harmful to NOX activity compared with dissolved O_2_.

#### Protein Concentration Change during Bubbling

**Figure 6:**
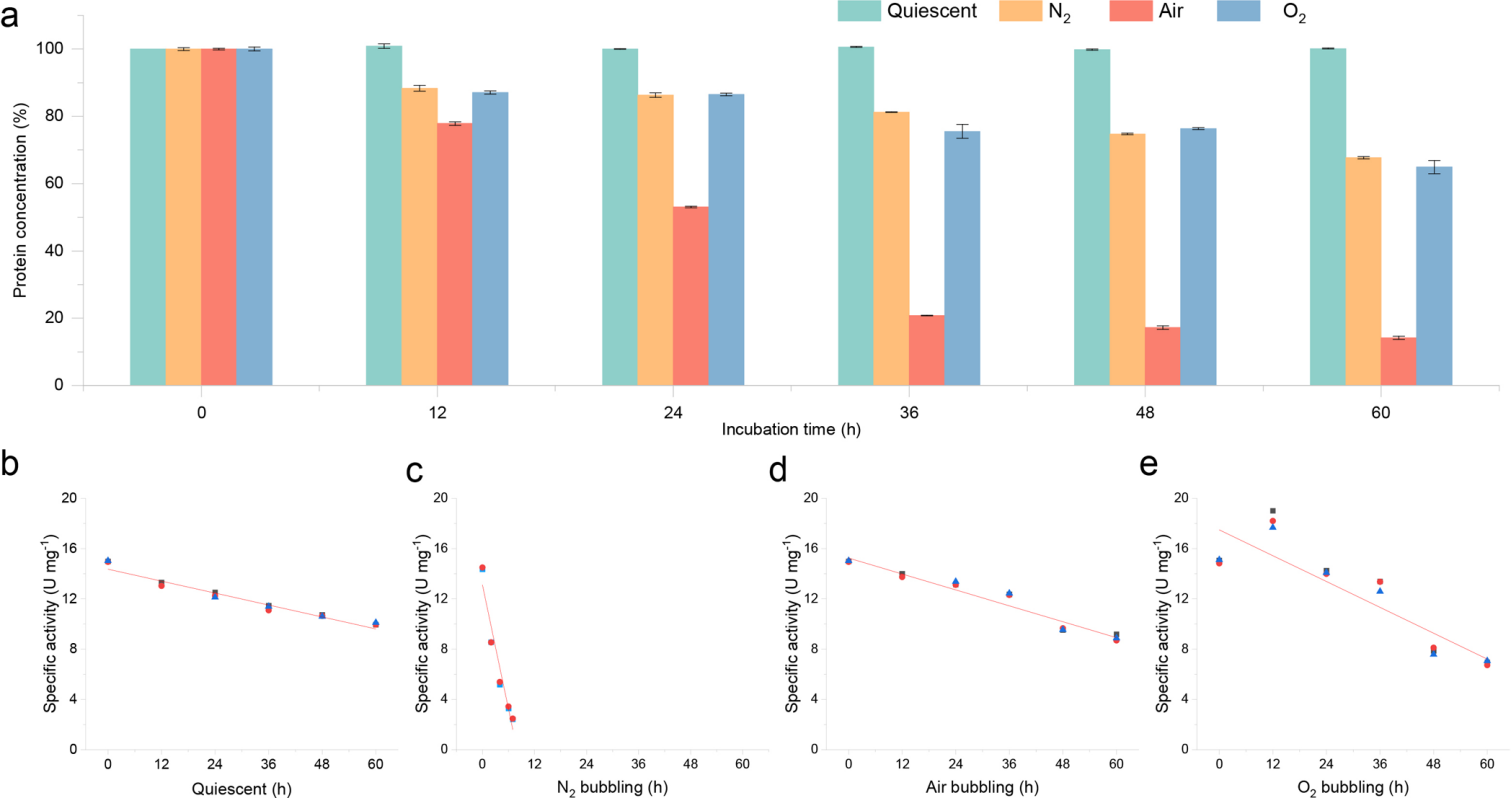
Protein concentration change of NOX solution in the bubble column under N_2_, air, and O_2_ bubbling (a) and the corresponding specific activity of NOX change with 0.15 L min^–1^ gas flow rate, 200 mL NOX solution, and 25 °C. The fitting equations of the decrease of the NOX specific activity in figures are (b) *y* = −0.079*x* + 14.361, *R*^2^ = 0.95; (c) *y* = −1.644*x* + 13.111, *R*^2^ = 0.94; (d) *y* = −0.106*x* + 15.244, *R*^2^ = 0.95; and (e) *y* = −0.171*x* + 17.493, *R*^2^ = 0.83.

The protein concentration of the NOX solution was measured under quiescent conditions and also gas bubbling, resulting in a decrease during gas bubbling (Tables S1–S4). The result is shown in Figure 6a. Under quiescent conditions, there was no decrease of protein concentration, but the specific activity of NOX decreased as expected (Figure 6a,b). The fitting equation of the NOX specific activity decrease was *y* = −0.079*x* + 14.361 (*R*^2^ = 0.95), and it was related to the natural deactivation of NOX in solution. In a bubble column, it was clear that the protein concentration decreased gradually under exposure to the gas–liquid interface. The fastest decrease of protein concentration occurred with air bubbling from 100 to 14% after 60 h with 0.15 L min^–1^ gas flow rate at 25 °C (Figure 6a). With N_2_ and O_2_ bubbling, the protein concentration decreased from 100 to 68% and 65%, respectively.

However, the specific activity of NOX decreased the fastest over a short N_2_ bubbling time compared with air bubbling and O_2_ bubbling (Figure 6c–e). The corresponding fitting equation was *y* = −1.644*x* + 13.111 (*R*^2^ = 0.94), where the decreasing slope was 21-fold, 16-fold, and 10-fold faster than quiescent conditions, air bubbling, and O_2_ bubbling. This result indicated that the dissolved N_2_ caused NOX deactivation rapidly and further confirmed that N_2_ might occupy the oxygen channel of NOX. This NOX with less activity (or total loss of activity) was still dissolved in solution and resulted in a similar protein concentration decrease compared with O_2_ bubbling (Figure 6a). However, decreasing NOX specific activity with O_2_ bubbling might be mainly caused by the over-oxidation of the NOX amino acid residue because the dissolved O_2_ concentration was much higher compared with air-saturated solution.^28^ The fitting equation of the specific activity decrease with O_2_ bubbling was *y* = −0.171*x* + 17.493 (*R*^2^ = 0.83) (Figure 6e). It should also be mentioned that the impurity protein in NOX solution might also be responsible for the protein concentration decreasing, which could also affect the specific activity.

In addition, the decreasing slopes of the NOX specific activity under quiescent conditions and air bubbling had a similar trend and were 0.079 and 0.106, respectively (Figure 6b,d), but the protein concentration under air bubbling decreased fast. It might indicate that the air–water interface only caused protein aggregation, and then the protein aggregation formed an insoluble precipitate and left the aqueous solution. The dissolved gases (N_2_ and O_2_) at this concentration had a limited effect on enzyme activity. It might also be the interface that caused a large decrease in NOX activity and meanwhile led to a decrease in the protein concentration, which also showed a similar decrease of the NOX specific activity under quiescent conditions and air bubbling (Table S5). Nevertheless, it was still suggested that O_2_ was more suitable for gas bubbling to the bubble column for NOX-catalyzed cofactor regeneration because of the high total activity of NOX under O_2_ bubbling during 60 h incubation. However, NOX was always used in cascade reactions together with dehydrogenase, and the stability of these dehydrogenases and cofactors has rarely been studied. Though the oxygen mass transfer coefficient *k*_L_*a* would be increased with pure O_2_ bubbling, most of these dehydrogenases dissolved in solution might be sensitive to high concentrations of dissolved O_2_, which might cause serious damage to enzyme activity. Moreover, some specific substrates might also be sensitive to high O_2_ concentrations. Likewise, if NOX has a high affinity toward O_2_ with a low *K*_MO_, which could be achieved by enzyme engineering, air bubbling would be more suitable and efficient to supply O_2_ for biocatalytic oxidation.

**Figure 7:**
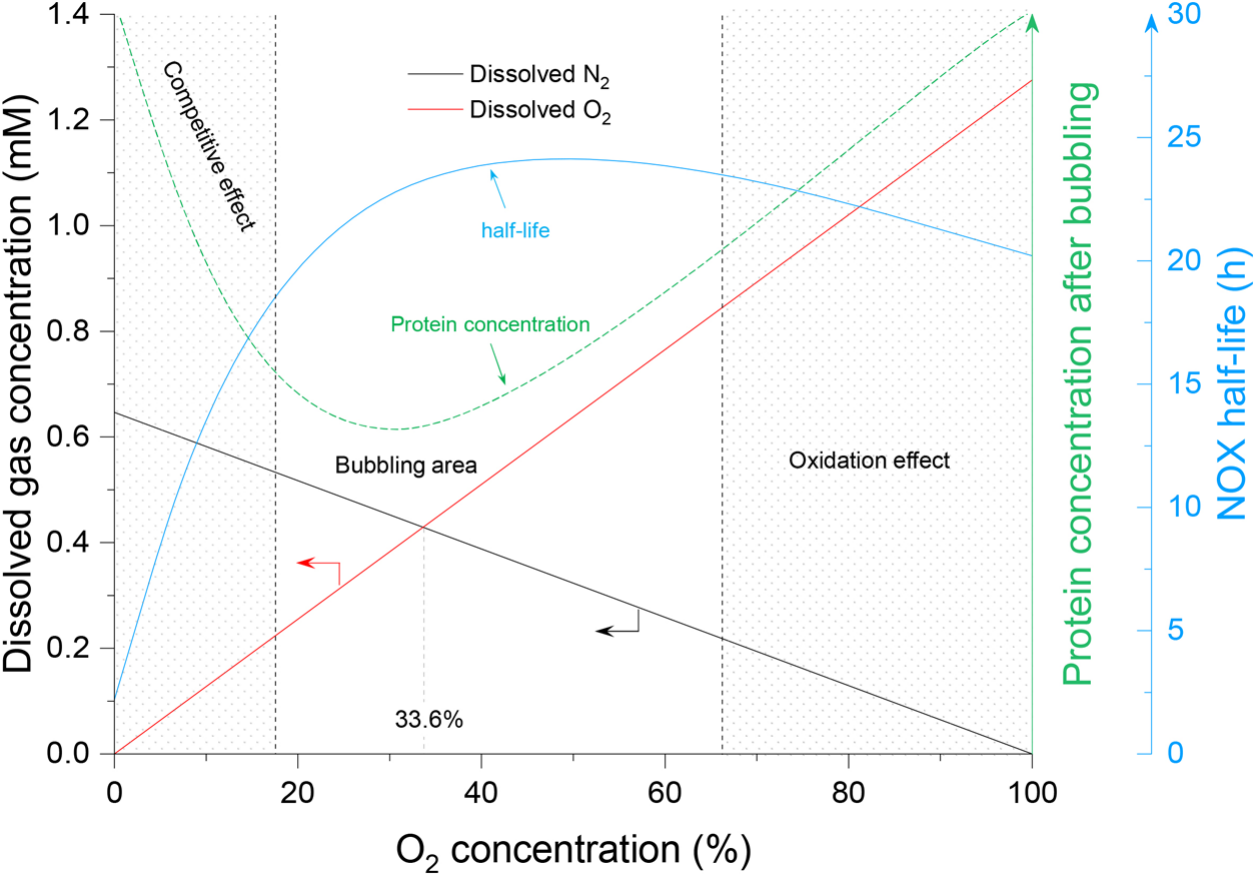
Operation map of the bubble column with the relationship between O_2_ concentration and protein aggregation, NOX half-life. Three areas were distinguished by the shadow and blank areas, and it showed the different effects of the NOX deactivation process. Competitive effect: N_2_ concentration was much higher than the O_2_ concentration, and N_2_ could easily get into the O_2_ channel of NOX (N_2_ effect area); bubbling area, the protein concentration loss occurred less compared with another two areas; oxidation effect: the high O_2_ concentration caused over-oxidation of the amino acid residue (O_2_ effect area).

Based on the above, an operation map of the bubble column for gas bubbling is illustrated in Figure 7. The relationship between the O_2_ concentration and protein concentration loss was also described; that is, protein concentration loss occurred less with N_2_ bubbling and O_2_ bubbling, but more protein concentration loss appeared with air bubbling. Meanwhile, three different areas were distinguished competitive effect area, bubbling area, and oxidation effect area (Figure 7). In the competitive effect area, N_2_ could easily get into the O_2_ channel of NOX, which caused fast enzyme deactivation (N_2_ effect area). In the bubbling area, the loss of protein concentration occurred less compared with another two areas. In the oxidation effect area, the high O_2_ concentration might cause over-oxidation of the amino acid residue, which was regarded as the reason for enzyme deactivation (O_2_ effect area). The half-life of NOX generated in this study is also involved in this figure, and it is 2.2, 28.6, and 20.2 h under N_2_, air, and O_2_ bubbling, respectively. The figure might possibly be a reference for the bubble column operation conditions. It indicated that the suggested O_2_ concentration for the NOX reaction was in a range of 50–70%. In this range, the effects of N_2_ (competitive effect) and O_2_ (oxidation effect) were reduced, the protein concentration after bubbling was relatively high, and less protein loss occurred. Meanwhile, a higher oxygen concentration in the supplied gas was more efficient for oxygen mass transfer. However, it might have more requirements about gas flow control for different N_2_ and O_2_ ratios to get the suitable O_2_ concentration. Further experiments could be conducted to determine more specific oxygen concentrations. The studies of enzymes and cofactors, which are involved in the cascade reaction together with NOX (e.g., oxidoreductases, natural and artificial cofactors), are also important to determine appropriate processes for biocatalytic oxidation.

#### Enzyme Structure Analysis

**Figure 8:**
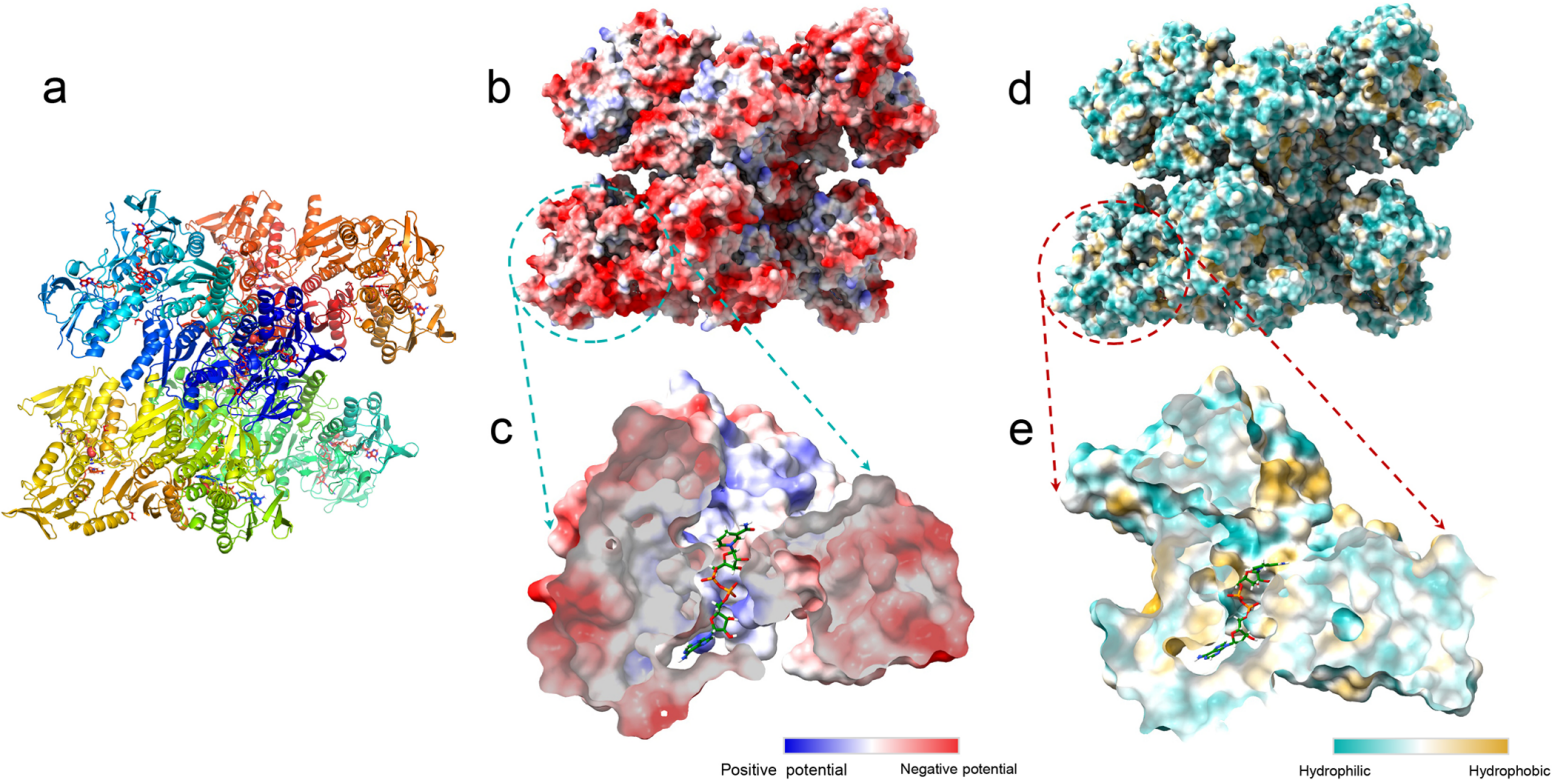
Surface property of water-forming NOX (PDB: 5VN0) with (a) 3D structure view, (b) electric potential distribution, and (c) corresponding cross-sectional view of the active site bound to NADH, (d) hydrophobic/hydrophilic distribution, and (e) corresponding cross-sectional view of the active site bound to NADH. Diagrams generated using UCSF ChimeraX (Version 1.4).

The NOX surface properties, including the electric potential and hydrophobicity/hydrophilicity, were analyzed by UCSF ChimeraX (*Version 1.4*) to further explain the results of NOX deactivation (Figure 8b,d). The cross section of the NOX active site is also shown in Figure 8c,e. The electric potential distribution of NOX showed that the NOX surface potential was more negatively charged (Figure 8b), but the active site was more positively charged according to the cross-sectional view of the active site (Figure 8c). It has been reported that the bubbles in aqueous solution carry a surface charge and are negatively charged due to excess OH^-^ groups at the corresponding gas–liquid interface.^29^ Therefore, a positively charged active site is more easily adsorbed on the gas–liquid interface, which might be the possible driving factor for NOX adsorption and the following aggregation at the gas–liquid interface. Then, the enzyme loses its activity due to the change of the three-dimensional structure and the shape of the active site. In Figure 8d, the surface of NOX was more hydrophilic. On the contrary, the hydrophobic region appeared in the inner part of NOX, especially in the active site (Figure 8c). This is also one of the factors that drive enzyme deactivation because of the hydrophobicity of the gas–liquid interface.

## Conclusions

Although enzyme deactivation has been known for a long time, the effects of pH, temperature, and solvents on enzyme stability are often studied in quiescent conditions. There are few studies that focus on the operational stability of enzyme, such as exposure to the gas–liquid interface and on the kinetic deactivation process of enzyme during operation. In this study, the kinetic stability of NOX was studied in a bubble column with different gases bubbling for 60 h, and the deactivation process was detected from 100% activity to less than 20% residual activity. It was found that NOX unfolding and aggregation at the gas–liquid interface resulted in the pH increase. The protein concentration decreased upon exposure to the gas–liquid interface, with most aggregation occurring with air bubbling compared with N_2_ and O_2_ bubbling. A high O_2_ concentration might cause over-oxidation of the amino acid residue. The deactivation of NOX under argon and N_2_ bubbling were very fast and had similar decreasing trends. It was found that the dissolved N_2_ in solution caused fast deactivation of NOX compared with the N_2_–water interface, which was confirmed by bubbling N_2_ and O_2_ alternately and also the comparison of the NOX specific activity in quiescent conditions and bubble column with N_2_, air, and O_2_ bubbling. Furthermore, the air–water interface had less effect on enzyme activity but caused serious protein loss from solution. A range of 50–70% O_2_ concentrations were suggested for NOX reaction operation because of the less protein concentration loss and long half-life of NOX according to the operation map of the bubble column generated in this study.

These results help our understanding of the kinetic deactivation process of NOX. According to this result, enzyme engineering might be inspired by the fact that only focusing on enhancing the affinity of the substrate and the thermal stability might not be enough. More attention should be paid to the kinetic stability of enzymes exposed to different operation conditions in biocatalytic oxidation process.

## Supporting Information Available

Absorbance decreasing of NADPH solution catalyzed by NOX; the standard curve of protein concentration; data of the protein concentration change in quiescent condition and in a bubble column with N_2_, air, and O_2_ bubbling; and the relationship of the NOX specific activity to activity and protein concentration under quiescent and bubbling conditions (PDF)

## Abbreviations

NAD(P)H: reduced nicotinamide adenine dinucleotide (phosphate)
NAD(P)^+^: nicotinamide adenine dinucleotide (phosphate)
NOX: NAD(P)H oxidase
ADH: alcohol dehydrogenase

## Nomenclature

*k*_L_*a*: oxygen mass transfer coefficient (h^–1^)
*K*_MO_: affinity constant of oxidase toward oxygen (mM)
*p*_gas_: gas phase partial pressure (kPa)
*H*: Henry’s law constant (kPa)
*C*: solubility of dissolved gas in water (M)
*k*_d_: enzyme deactivation kinetic constant (h^–1^)
*a*: enzyme residual activity (mM min^–1^)
*a*_0_: enzyme original activity (mM min^–1^)
ρ: liquid density (kg m^–3^)
