# Supplementary material for: Effect of Nitrogen, Air, and Oxygen on the Kinetic Stability of NAD(P)H Oxidase Exposed to a Gas–Liquid Interface
Source: Org Process Res Dev. 2023 May 19;27(6):1111–21. doi: 10.1021/acs.oprd.3c00095 (PMC11108306; doi:10.1021/acs.oprd.3c00095)
Supplement: Supplementary file 1 — op3c00095_si_001.pdf [file op3c00095_si_001.pdf]

Effect of Nitrogen, Air and Oxygen on the Kinetic Stability of NAD(P)H Oxidase Exposed to Gas-Liquid Interface

Jingyu Wang[1], Elif Erdem[1], John M. Woodley[1,*]

[1]Department of Chemical and Biochemical Engineering, Technical University of Denmark, 2800 Kgs. Lyngby, Denmark

[*]Corresponding author: John M. Woodley (jw@kt.dtu.dk)

Table of Contents

Figure S1	*The absorbance decreasing of NADPH solution catalyzed by NOX*
Figure S2	*The relationship between protein concentration and the absorbance of NOX solution mixed with Coomassie Plus™ protein assay reagent*
Table S1	*Protein concentration change in quiescent condition*
Table S2	*Protein concentration change in bubble column with N_2 bubbling*
Table S3	*Protein concentration change in bubble column with air bubbling*
Table S4	*Protein concentration change in bubble column with O_2 bubbling*
Table S5	*The change of NOX specific activity to activity and protein concentration under quiescent and bubbling condition*

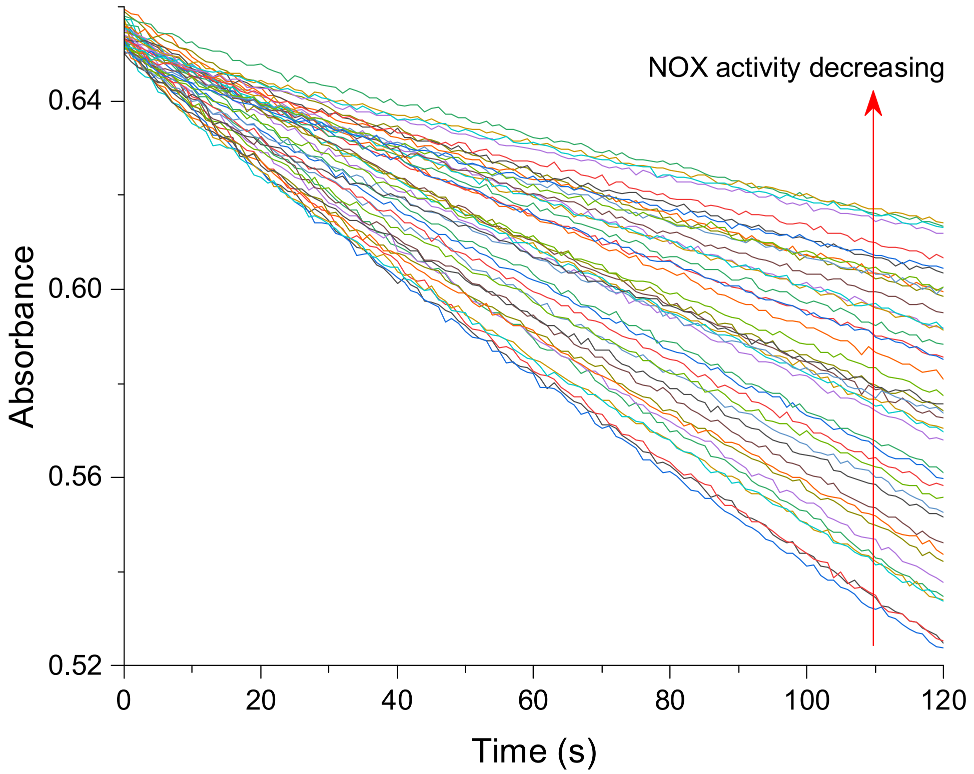

Figure S1 The absorbance decreasing of NADPH solution catalyzed by NOX. The NOX solution was taken from bubble column after different time air bubbling with 0.05 L min^{-1} gas flow rate (200 mL NOX solution in bubble column). The slope is related to the activity of NOX according to Beer-Lambert law. The extinction coefficient of NADPH at 340 nm is ε_{340}=6.22 mM^{-1} cm^{-1}.

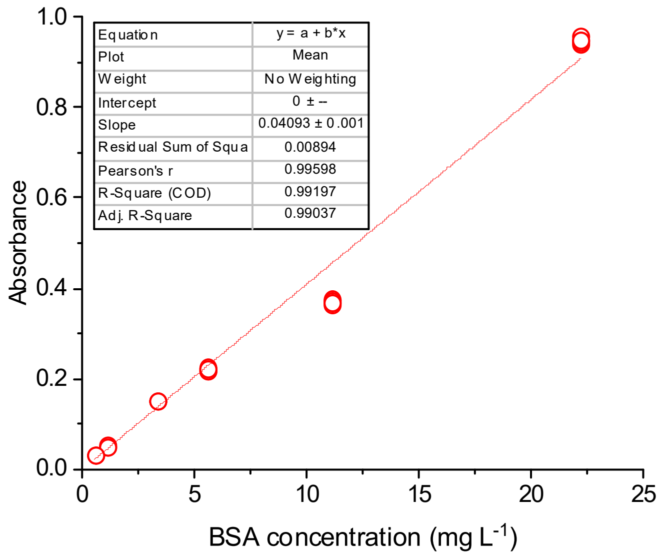

Figure S2 The relationship between protein concentration and the absorbance of NOX solution mixed with Coomassie Plus™ protein assay reagent at 595 nm. The corresponding fitting equation was: y=0.04093x, R^2=0.99.

Table S1 Protein concentration change in quiescent condition (measured at 595 nm with UV/Vis spectra)

Incubation time (h)	Absorbance		
0	0.641	0.641	0.641
12	0.644	0.65	--
24	0.642	0.641	0.641
36	0.646	0.645	0.644
48	0.639	0.641	0.64
60	0.642	0.643	0.641

Table S2 Protein concentration change in bubble column with N_2 bubbling (measured at 595 nm with UV/Vis spectra)

Incubation time (h)	Absorbance		
0	0.617	0.62	0.615
12	0.551	0.545	0.54
24	0.531	0.53	0.538
36	0.501	0.502	0.501
48	0.463	0.462	0.46
60	0.418	0.416	0.42

Table S3 Protein concentration change in bubble column with air bubbling (measured at 595 nm with UV/Vis spectra)

Incubation time (h)	Absorbance		
0	0.641	0.641	0.641
12	0.518	0.518	--
24	0.487	0.488	0.484
36	0.442	0.442	0.444
48	0.262	0.261	0.263
60	0.103	0.106	0.104

Table S4 Protein concentration change in bubble column with O_2 bubbling (measured at 595 nm with UV/Vis spectra)

Incubation time (h)	Absorbance		
0	0.630	0.635	0.637
12	0.549	0.555	0.553
24	0.551	0.546	0.549
36	0.466	0.479	0.492
48	0.482	0.485	0.485
60	0.403	0.406	0.423

Table S5 The change of NOX specific activity to activity and protein concentration under quiescent and bubbling condition

Equation		Unit	Quiescent	Bubbling*
Specific activity =	a	U	↓	↓↓
	c_{NOX}	mg	——	↓

*The decreasing of NOX specific activity under gas bubbling has the similar slope to quiescent, the possible change of NOX activity (a) and c_{NOX} were ↓↓ (more decreasing) and ↓ (decreasing), respectively.
